# *Lactobacillaceae* and Parkinson's disease: An apparent paradox

**DOI:** 10.1177/1877718X241312401

**Published:** 2025-01-29

**Authors:** Marieke ME van der Maden, Marcel M Verbeek, Milan Beckers

**Affiliations:** 1 Faculty of Science, Radboud University, Nijmegen, The Netherlands; 2 Department of Neurology, Donders Institute for Brain, Cognition and Behaviour, Radboud University Medical Center, Nijmegen, The Netherlands; 3 Radboudumc Centre of Expertise for Parkinson & Movement Disorders, Nijmegen, The Netherlands; 4 Department of Human Genetics, Radboud University Medical Center, Nijmegen, The Netherlands

**Keywords:** Parkinson's disease, microbiota, Lactobacillaceae, levodopa

## Abstract

Parkinson's disease (PD) is a neurodegenerative disorder predominantly known for its motor symptoms such as bradykinesia, rigidity and tremor, but the disorder is also increasingly recognized for its association with impaired gastrointestinal function. The composition of the gut microbiome is known to be different in PD compared with healthy individuals. One of the bacterial families with increased abundance in people with PD is *Lactobacillaceae*. Interestingly, opposite effects have been ascribed to *Lactobacillaceae* in PD. A number of studies have linked *Lactobacillaceae* spp. in the gut to worse motor function, and to premature degradation of levodopa. However, other studies have linked administration of *Lactobacillaceae*-containing probiotics to improved motor function and reduced gastrointestinal problems. In this narrative review, we investigate this apparent paradox. The key to its understanding appears to lie in the specific species of *Lactobacillaceae*. The species *L. plantarum* in particular seemed to show a correlation with improved motor symptoms, as well as a reduction in intestinal inflammation, whereas *L. brevis, L. curvatus* and *L. fermentum* have properties that might be detrimental to people with PD.

## Plain language summary

Parkinson's disease (PD) is an illness of the brain, best known for its problems with movement, such as stiffness, slowness, and shaking. However, it is becoming ever more recognized that it also comes with problems of the stomach and bowels. The composition of the gut flora (intestinal microbiome) is different in PD compared to healthy persons. *Lactobacillaceae* is a bacterial family that is present in larger amounts in the bowels of people with PD. Interestingly, researchers have described opposite effects for *Lactobacillaceae* in PD. On the one hand, some scientific studies have found that these bowel bacteria are connected to worse movement problems, and with early breakdown of the PD medicine levodopa. On the other hand, there are studies that found that treating people with PD with probiotics containing *Lactobacillaceae* improved their movement and lessened their stomach and gut problems. For this article, we reviewed the various studies into this subject. We found that the effect seems to depend on the particular species of bacterium, within the wider *Lactobacillaceae* family. The species *L. plantarum* shows favorable properties in PD: fewer problems with movement, and less inflammation of the bowels. The species *L. brevis*, *L. curvatus* and *L. fermentum*, on the other hand, have properties that might be negative for people with PD.

## Introduction

Parkinson's disease (PD) is the fastest growing neurodegenerative disorder, with approximately 6.1 million people affected worldwide in 2016, and global prevalence expected to exceed 12 million by 2040.^1^

In recent years, a growing body of research has explored the connection between the intestinal tract and the brain in PD, the so-called gut-brain axis. Intestinal microorganisms play a central role in this theoretic framework. Intriguingly, *Lactobacillaceae*, one of the bacterial families that has been most frequently associated with altered gut-brain interaction in PD, seems to play a dualistic role here. On the one hand, the use of particular strains of *Lactobacillaceae* as probiotics has been associated with improvement in both motor and non-motor symptoms.^2^ On the other hand, and seemingly paradoxical, *Lactobacillaceae* is also a family of which an increased abundance in PD has been correlated to worse motor symptoms.^3,4^ In this review, we present a possible explanation for this apparent paradox.

## The connection between gut pathology and brain pathology in PD

The pathophysiological hallmark of PD is the degeneration of dopaminergic neurons in the substantia nigra (SN), leading to abnormalities of movement, behavior and emotions.^5^ This degeneration appears to be caused by the formation of intraneuronal aggregates of the protein alpha-synuclein, so-called Lewy bodies (LBs). They can be found in various brain regions of people with PD, and are believed to play a pathological role in the progression of the disease.^6^ In recent years, accumulating clinical and pathological evidence has shown that LBs can be demonstrated in enteric nerves before they appear in the brain, possibly spreading through the vagus nerve towards the brainstem.^7–9^ Multiple studies, performed in animal models as well as in humans, have shown that bidirectional communication makes it possible for LBs to spread from enteric neurons, through the vagus nerve, into the brain, following a prion-like spreading cascade.^10^ Still, it has not been proven that the pathology actually starts in the intestinal tract.^11^

Seminal work by Scheperjans et al. in 2014 described a correlation between PD and altered gut microbiota.^12^ This was supported by differences in the abundance of several genera of bacteria, when comparing fecal samples of people with PD with those of healthy individuals. These differences were found even when accounting for factors such as diet or gastrointestinal symptoms. This suggests that they are driven by the disease itself, and not by lifestyle or environmental factors.^13^ However, these factors are notoriously difficult to correct for, and to date the direction of causality between gut microbiota alterations and PD is still uncertain. *Lactobacillaceae* is one of the bacterial families of which abundance is increased in PD.^14^

An altered gut microbial composition in PD has also been linked to a pro-inflammatory state of the intestines. A study in 38 people with PD and 34 healthy controls showed that the gut of people with PD contained a significantly higher abundance of putative pro-inflammatory bacteria.^15^ Another study found an increase in mRNA transcripts encoding pro-inflammatory cytokines, as well as glial markers, when analyzing colonic biopsies of people with PD and comparing them with healthy controls.^16^ Lastly, a study found high levels of stool immune factors in fecal samples of people with PD, suggesting the presence of intestinal inflammation.^17^ A relationship between intestinal inflammation and pathogenic alpha-synuclein accumulation has been shown in literature before.^18^ Therefore, it can be hypothesized that the inflammatory environment plays a role in the pathogenesis of PD.

## Gut microbial dysbiosis and PD

The gut microbiota are defined as all the living microorganisms found in the intestines.^19^ When the composition or function of the microbiota is altered in a way that facilitates pathological processes, it is referred to as intestinal dysbiosis. This has been linked to various diseases, such as gastrointestinal, autoimmune and neurological diseases.^20^ In multiple studies on dysbiosis in PD, performed in animal models as well as in humans, alterations in the composition of both fecal and mucosal microorganisms were found.^21^ Although, in part due to heterogeneity of study designs, a wide variety of alterations in microbiota composition has been reported and it is not yet possible to define a PD “fingerprint” in gut microbiota, a meta-analysis concluded that increased abundance of, among others, *Lactobacillaceae* was consistent through several studies done on the gut microbiota in people with PD.^22^

*Lactobacillaceae* are rod-shaped, gram-positive, non-spore-forming, facultative anaerobic bacteria of the phylum Firmicutes. The relationship between *Lactobacillaceae* and their host is mutualistic, with *Lactobacillaceae* providing help in digestion, provision of supplementary nutrients and protection from pathogens, and the host offering accommodation and nutrients.^23^ *Lactobacillaceae* fall under the category of short-chain fatty acid (SCFA)-producing bacteria. SCFAs are bacterial metabolites that are produced by fermentation of non-digestible carbohydrates, such as ethanoic acid, propanoic acid, butanoic acid and lactic acid, and play an important role in maintaining the intestinal barrier integrity, production of mucus and protection against pathogens, toxins and endogenous substances.^24–26^ They are also capable of modulating anti-inflammatory processes via multiple pathways.^27–29^

Another type of bacterial metabolites often discussed in the context of dysbiosis are lipopolysaccharides (LPS). These are endotoxins exclusively produced by gram-negative bacteria. There are several arguments to hypothesize a role of LPS in the pathogenesis of PD.^30–35^ However, since *Lactobacillaceae* are gram-positive bacteria, these reasons will not be discussed further in this review.

A causal link between gut microbial dysbiosis and the development of PD has not yet been proven. There are two prominent hypotheses: one is that the differences in composition of the microbiota of people with PD as compared to healthy individuals are a pathophysiological trigger for the development of PD. The other, conversely, considers the altered gut microbiota as a consequence of PD. In addition, certain aspects of gut dysbiosis have been linked to specific disease symptoms and to the effect of antiparkinson medication. This latter aspect will be further explored in the following sections.

## *Lactobacillaceae* spp. in the gut of people with PD

Specific *Lactobacillaceae* species are increased in abundance in the gut of people with PD, including *L. brevis*, *L. casei*, *L. fermentum*, *L. gasseri*, *L. reuteri*, *L. ruminis* and *L. mucosae*.^36,37^ In contrast to these findings, increased abundance of *Lactobacillaceae* has never been detected in Chinese studies.^38^ These studies suggest that, although based on a very limited number of publications, these differences might be caused by genetic and environmental differences in the studied individuals, including differences in diet or lifestyle, which might lead to the observed discrepancies. It should be noted that the sample size of the Chinese study was small, limiting the significance of these results.

A direct link has been suggested between *Lactobacillaceae* spp. and alpha-synuclein secretion.^12^ This was substantiated by multiple studies. In animal studies, it was shown that administration of *L. rhamnosus* increases the firing rate of vagal afferents as well as cortical neurons.^39–41^ Additionally, it has been found that *L. reuteri* administration increased the firing rate as well as the excitability of enteric neurons in rats.^42^ In mice and rat studies, increased activity of enteric neurons as well as cortical neurons influences secretion of alpha-synuclein, of which accumulation and aggregation is related to PD pathogenesis.^43,44^ As stated before, the species *L. reuteri* has been found to be increased in the gut of individuals with PD, while *L. rhamnosus* has not.

**Figure 1.**
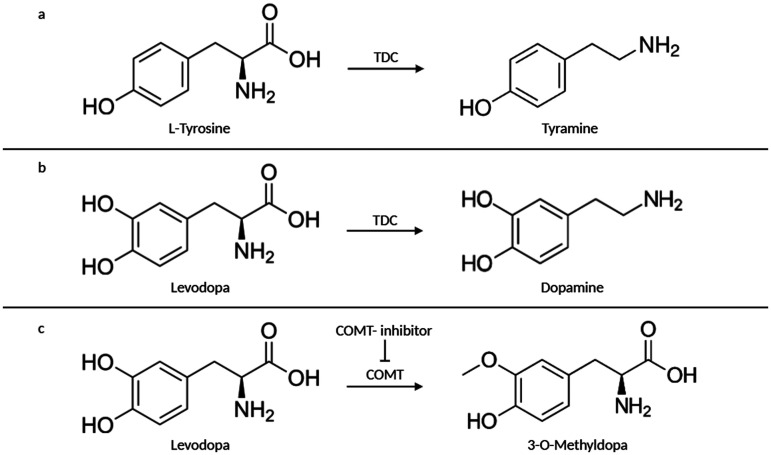
(a) Tyrosine decarboxylase (TDC) decarboxylates L-tyrosine into tyramine (b) TDC is also capable of decarboxylating levodopa into dopamine (c) Catechol-*O*-methyltransferase (COMT) inhibitors prevent transmethylation of levodopa to 3-*O*-methyldopa.

The *Lactobacillaceae* species *L. brevis, L. curvatus* and *L. fermentum* are capable of producing the enzyme tyrosine decarboxylase (TDC).^45^ The function of this enzyme is to decarboxylate L-tyrosine into tyramine (Figure 1(a)).

However, it also shows ‘promiscuous activity’ towards levodopa, decarboxylating it into dopamine, due to the high similarity in chemical structure of these molecules* (Figure 1(b)).*^46^

To be effective, levodopa must cross the intestinal mucosa and blood-brain barrier (BBB) and subsequently be converted into dopamine by the enzyme aromatic *L*-amino acid decarboxylase (AADC). However, if gut microbiota, such as the aforementioned *Lactobacillaceae* species*,* metabolize levodopa before it is able to cross the intestinal mucosa, the bioavailability of the medication is reduced, rendering it less effective. This is one of the pathways of the phenomenon referred to as peripheral levodopa resistance.^47^ Two of these TDC-producing species, namely *L. brevis* and *L. fermentum*, show increased abundance in the gut of people with PD. As mentioned before, it is not certain if the altered gut microbiota are the trigger for PD, or a consequence of PD. Similarly, it remains to be investigated to what extent the increase in TDC-producing bacteria is enabled by the increased presence of substrate (oral levodopa). It has been hypothesized that prolonged levodopa treatment favors the growth of TDC-producing bacteria.^48^ However, another study concluded that levodopa does not have an effect on the gut microbiota composition.^36^

Catechol-*O*-methyltransferases (COMT) are enzymes capable of metabolizing levodopa by transmethylation to 3-*O*-methyldopa, reducing its bioavailability. COMT inhibitors are often used as an adjunct therapeutic together with levodopa, prolonging the clinical effect of levodopa in people with PD (Figure 1(c)).

A meta-analysis found a correlation between COMT inhibitor intake and an increased abundance of *Lactobacillaceae* spp. in the gut.^24^ Additionally, another study found that COMT inhibitors influenced the abundance of several intestinal bacterial species, including an increase in *Lactobacillaceae* spp. abundance.^49^ The mechanism behind this is still unknown. COMT inhibitors can have adverse effects, such as diarrhea, and prolonged diarrhea might lead to alteration in the gut microbiota.^50,51^ However, there is no indication in literature that only COMT inhibitor users who suffer diarrhea have altered gut microbiota. Reverse causality can also be considered: an increase of the *Lactobacillaceae* species *L. brevis, L. curvatus* and *L. fermentum* might worsen motor function, as TDC produced by those species can cause reduced levodopa bioavailability^47^; the associated shortened duration of action of levodopa might then prompt the treating physician to initiate COMT inhibitor therapy.

In conclusion, it can be said that the abundance of *Lactobacillaceae* spp. is associated with the presence of PD and the efficacy of levodopa treatment. However, the exact mechanisms behind this, and the direction of any causality, are still unclear.

## *Lactobacillaceae* spp. in relation to motor and non-motor symptoms in PD

A 2022 study found that increased abundance of *Lactobacillaceae* in the gut of people with PD was associated with motor complications.^3^ Wearing-off was found to be an independent factor associated with an increase in relative abundance of intestinal *Lactobacillaceae*. This increase in wearing-off could be speculated to be caused by decreased levodopa bioavailability as a consequence of decarboxylation into dopamine by TDC-producing bacteria. Unfortunately, the 16S rRNA technique used in that study precludes identification of bacteria at the species level. Therefore, it cannot be ascertained which specific *Lactobacillaceae* species was correlated with wearing-off and whether it was indeed TDC-producing.

Another 2022 study also found a positive association between *Lactobacillaceae* and motor symptoms in people with PD.^4^ In this study, people with PD were divided into a group that received a probiotic containing *Bifidobacterium animalis* subsp. *lactis*, and a placebo-treated group. 97.91% of the probiotic group and 100% of the placebo group used levodopa at baseline. Comparisons of the microbiota between the groups at different time points revealed that, after treatment, the abundance of *L. fermentum* was decreased in the probiotic group, and that *L. fermentum* abundance was significantly and positively associated with a poorer mental state and more severe motor symptoms. Additionally, the study demonstrated that after one month of probiotic administration, serum dopamine levels increased, possibly suggesting an increase in levodopa available enterally for absorption through the intestinal mucosa. The relationship between the decreased abundance of *L. fermentum* in the probiotic group, the more severe motor symptoms associated with *L. fermentum* abundance and the elevated serum dopamine levels in the probiotic group might be explained by the ability of *L. fermentum* to decarboxylate levodopa into dopamine by producing TDC. Unfortunately, TDC activity was not measured in this study. The authors hypothesize instead that tyrosine hydroxylase (TH)-mediated conversion of *L*-tyrosine to levodopa might be responsible for the increased levodopa bioavailability and improved PD symptoms. A previous murine study into probiotic-driven alleviation of PD symptoms found increased TH staining in the substantia nigra of sacrificed mice.^52^

In addition to the motor symptoms, non-motor symptoms are also very common in PD. An example of this is the manifestation of gastrointestinal problems, which show a prevalence as high as 77% among people with PD.^53^ This can be even higher for specific symptoms such as gastroparesis, which has a prevalence ranging from 70% to 100%.^54^ Intestinal inflammation has been mentioned in multiple studies investigating the gut of people with PD, and this inflammatory environment might be involved in the pathogenesis of PD.^16–18^ However, the role of *Lactobacillaceae* specifically in relationship to PD-related gastro-intestinal symptoms has not been described in literature.

### Small-intestinal bacterial overgrowth

Small-intestinal bacterial overgrowth (SIBO) is a form of gut dysbiosis and is defined as increased bacterial density in the small intestine, caused by either proximal migration of colonic bacteria or by downstream migration of upper respiratory tract microflora.^55,56^ SIBO leads to symptoms such as bloating, abdominal pain or discomfort, diarrhea, and fatigue. The severity of the symptoms correlates with the degree of bacterial overgrowth as well as the extent of intestinal inflammation.^57^ It is thought that SIBO might cause an increase in intestinal permeability, also known as gut leakiness, and promote translocation of bacteria and endotoxins across the intestinal epithelium, which then induces an inflammatory response. Research suggests that intestinal inflammation can trigger microglial activation, amplifying neurodegeneration, which in turn leads to an increase in motor symptoms.^58^ A meta-analysis concluded that there is a strong association between SIBO and PD, with nearly half of people with PD testing positive for SIBO.^59^ *Lactobacillaceae* is one of the bacterial families that is present in excess in SIBO.^60^ Bacterial overgrowth has been linked to worse motor function in PD as well as to an increased intestinal permeability.^5,61–63^ In 2011, a study showed that people with PD exhibit a significantly greater intestinal permeability compared to healthy controls.^64^ This hyperpermeability correlated with oxidative stress and misfolded alpha-synuclein accumulation in the intestines. These studies together lend support to a hypothetical model in which gut dysbiosis leads to intestinal inflammation, which then triggers misfolding of alpha-synuclein.

### Ghrelin

An increase of *Lactobacillaceae* in the gut is correlated with a decrease in the gastrointestinal hormone ghrelin, which is also known as the “hunger hormone”.^65^ This hormone is produced in the stomach, and regulates gastric motility, gastric emptying, secretion of gastric acid and glucose metabolism.^66^ Ghrelin binds to growth hormone secretagogue receptors (GHSRs), which are found in several brain areas, including the substantia nigra. It has been shown that ghrelin plays a role in maintaining and protecting the normal nigrostriatal dopamine function and it might have a neuroprotective effect against dopaminergic degeneration in mice.^67–69^

Impaired ghrelin secretion as well as downregulation of GHSRs in PD was reported in multiple studies.^70–73^ This makes sense, as ghrelin levels and dopamine levels are in direct relation with each other. Ghrelin receptors are expressed on dopaminergic cells in the ventral tegmental area, stimulation of which results in increased dopamine release.^74,75^ It has been suggested that reduced ghrelin levels contribute to worse motor function in PD.^72^ Perhaps this has a pharmacokinetic origin: as reduced ghrelin levels lead to impaired gastric emptying, levodopa transit from stomach to small intestine may be hindered, leading to a reduced bioavailability of levodopa, ultimately resulting in worse motor function. A direct negative effect of decreased ghrelin levels on nigrostriatal dopamine function may be a viable alternative hypothesis.

**Figure 2.**
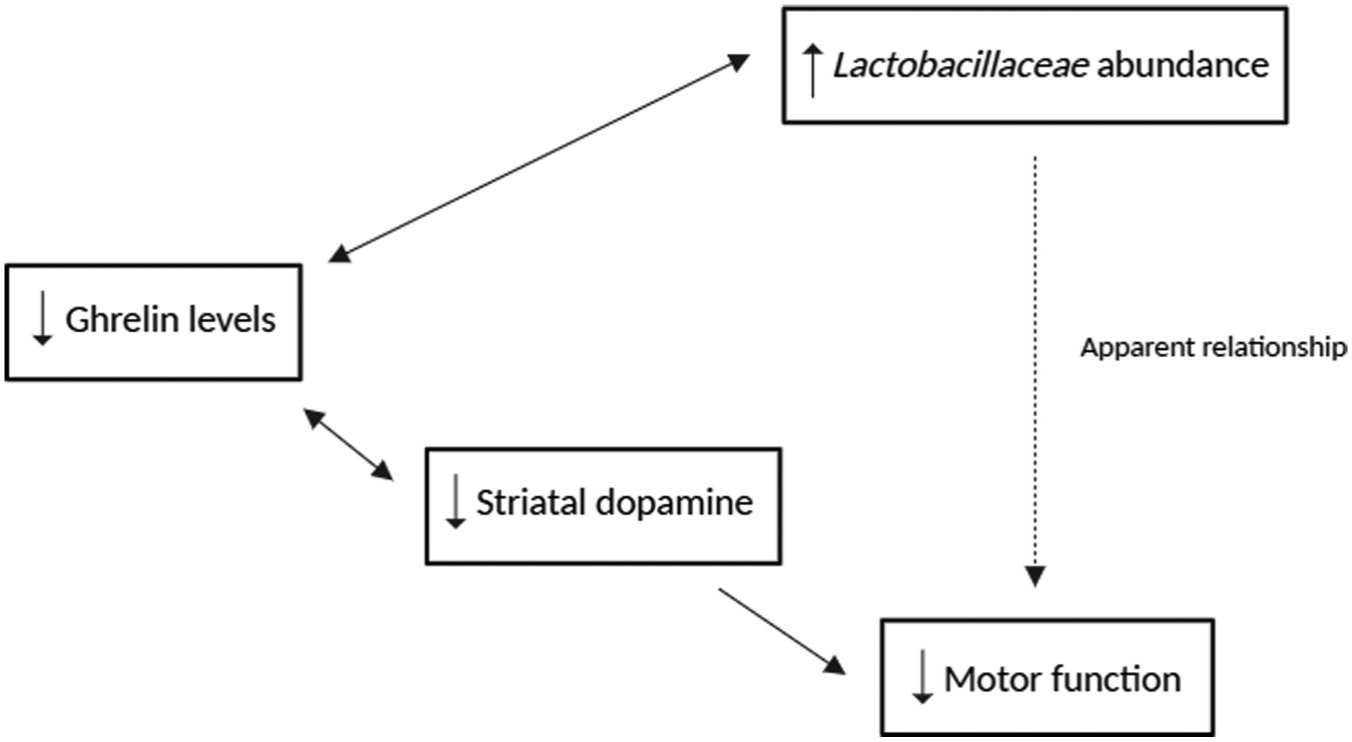
The apparent relationship between *Lactobacillaceae* spp., ghrelin levels and motor function, and their potential confounders.

A 2017 study found that changes in the microbiota can have a direct effect on plasma ghrelin levels. It was shown that eradication therapy for *H. pylori*-infection significantly decreased the concentration of active ghrelin in blood plasma.^76^ The reverse relation may also be true, namely that changes in plasma ghrelin levels have a (direct) effect on the microbiota, as has been shown with other hormones.^77–79^ Therefore, it can be hypothesized that the relative increase in *Lactobacillaceae* spp. in the gut of people with PD could be a direct result of the decreased ghrelin levels. In this hypothesis, ghrelin is a confounder for the apparent correlation between the increased abundance of *Lactobacillaceae* spp. and worse motor function. The link between reduced ghrelin levels and worse motor function could then have dopamine decrease as a confounder (Figure 2).

**Figure 3.**
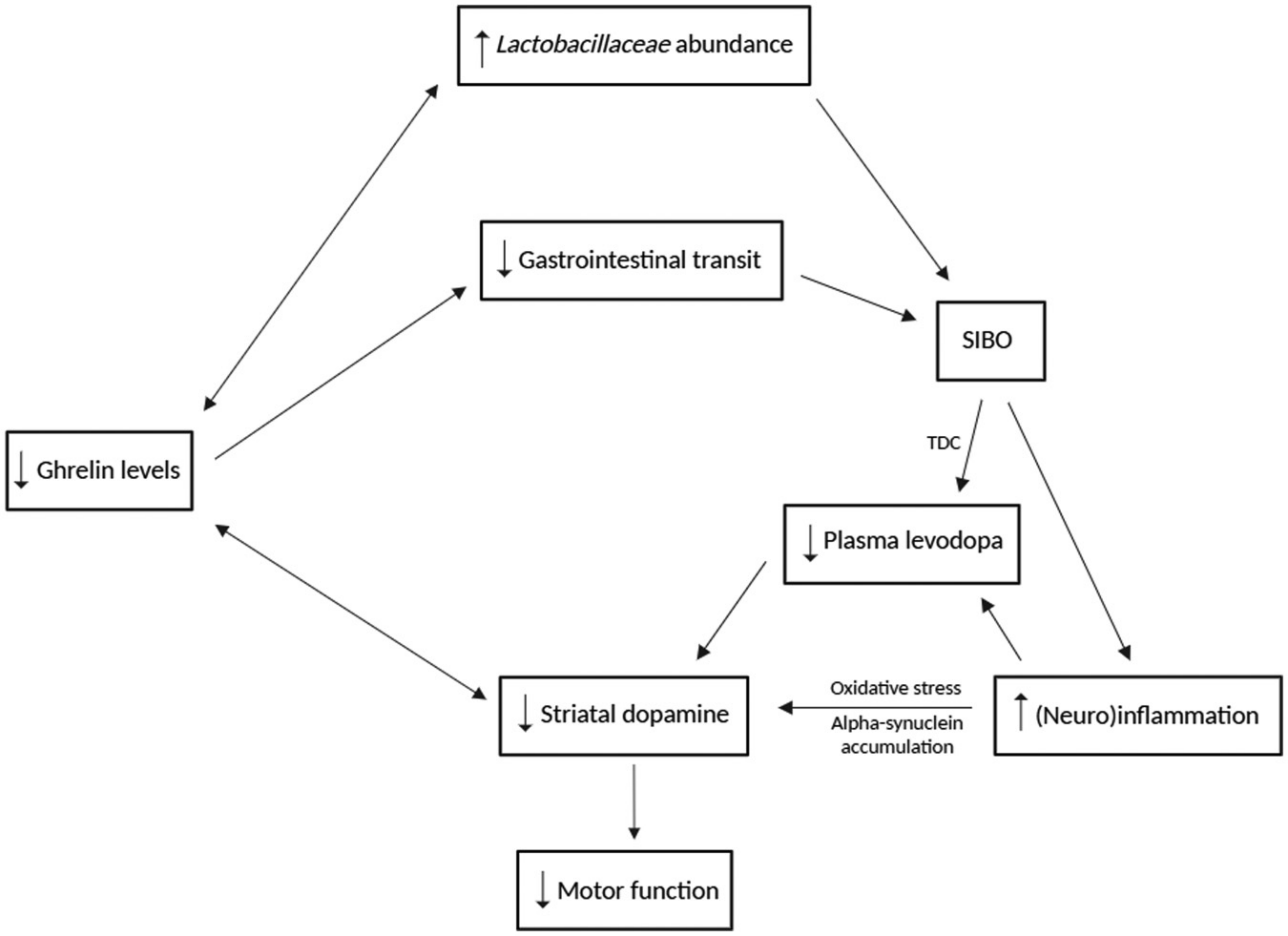
The possible mechanisms that may explain the relationship between *Lactobacillaceae* spp. and symptomatology in people with PD.

*Lactobacillaceae* spp. are thus correlated with the presence of motor as well as non-motor symptoms in PD. The possible mechanisms behind this are summarized in Figure 3.

## *Lactobacillaceae*-containing probiotics as a therapeutic agent for PD

Probiotics are defined as living microorganisms that, when administered in adequate amounts, confer health benefits to the host.^80^ Probiotics might have therapeutic use in PD, as it is thought that they are capable of altering the gut microbiota, which may reduce the severity of the gastrointestinal problems present in many of the people with PD.^81^ No serious side effects were described in clinical studies into probiotics for PD thus far, which might make them a safe treatment option.^82^

Several studies have been done in mouse models using various strains of *Lactobacillaceae* as a probiotic treatment. Many of the studies found that *Lactobacillaceae-*containing probiotics significantly improved motor function and decreased levels of pro-inflammatory factors and/or increased levels of anti-inflammatory factors.

In addition to studies done in mouse models, multiple clinical studies have been carried out, treating people with PD with *Lactobacillaceae*-containing probiotics. Studies investigating gastrointestinal problems found improved stool consistency, bowel habits and other gastrointestinal discomforts in people with PD. However, results varied. For instance, one study found that the probiotic administration caused a significant reduction of abdominal pain and bloating, while there was no effect on constipation.^83^

In addition to gastrointestinal problems, various studies have also investigated other effects of probiotics in people with PD. For instance, multiple studies found a downregulation of certain pro-inflammatory factors and/or upregulation of anti-inflammatory factors. Furthermore, two studies mentioned improvements in the Movement Disorder Society-sponsored revision of the Unified Parkinson's Disease Rating Scale (MDS-UPDRS) score. However, these improvements both did not cross the threshold for the minimal clinically important difference (MCID). The results of all the studies mentioned above are summarized in Table 1.

**Table 1.** Summary of probiotic treatment results in murine models and clinical trials.

Murine study	Probiotic	Model	Duration	Findings
Srivastav et al., 2019^52^	*B. animalis* subsp. *lactis*, *L. acidophilus* and *L. rhamnosus* GG	MPTP-induced PD in C57BL/6 mice and rotenone-induced PD in C57BL/6 mice	4 weeks	Reduced dopaminergic cell loss, increased levels of butyrate and neurotrophic factors, downregulation of MAO-B
Hsieh et al., 2020^84^	*B. bifidum*, *B. longum*, *L. l. lactis*, *L. plantarum* LP28, *L. rhamnosus* and *L. rhamnosus* GG	MitoPark PD mice	16 weeks	Improved gait patterns, balance function and motor coordination, reduced dopaminergic cell loss
Liao et al., 2020^85^	*L. plantarum* PS128	MPTP-induced PD in C57BL/6 mice	4 weeks	Improved motor function, decreased levels of IL-1β, IL-6 and TNF-α, corticosterone, increased levels of dopamine, norepinephrine, GSH and BDNF, reduced dopaminergic cell loss
Perez Visnuk et al., 2020^86^	*L. plantarum* CRL2130*, S. thermophilus* CRL807 and *S. thermophilus* CRL808	MPTP-induced PD in C57BL/6 mice	3 weeks	Improved motor function, decreased levels of IL-6 and TNF-α, increased levels of IL-10
Wang et al., 2021^87^	*L. plantarum* DP189	MPTP-induced PD in C57BL/6 mice	2 weeks	Improved motor function
Perez Visnuk et al., 2022^88^	*L. plantarum* 725 or *L. plantarum* CRL2130	MPTP-induced PD in C57BL/6 mice	3 weeks	Improved motor function, decreased levels of MCP-1, IL-6 and TNF-α, increased levels of IL-10, reduced dopaminergic cell loss
Wang et al., 2022^89^	*L. plantarum* DP189	MPTP-induced PD in C57BL/6 mice	2 weeks	Decreased levels of MDA, ROS, IL-1β, IL-6 and TNF-α, increased levels of SOD, GSH-Px and IL-10, reduced α-synuclein accumulation in SN
Chu et al., 2023^90^	*L. plantarum* CCFM405	Rotenone-induced PD in C57BL/6 mice	9 weeks	Improved motor function, decreased constipation and levels of IL-1β, IL-6 and TNF-α, increased levels of dopamine and BCAAs, normalized gut bacterial composition
Lee et al., 2023^91^	*L. plantarum* PS128	Rotenone-induced PD in C57BL/6 mice	6 weeks	Improved motor function, decreased levels of TNF-α, increased levels of dopamine, IL-10 and BDNF, reduced dopaminergic cell loss and microglial activation
Aktas et al., 2024^92^	*L. rhamnosus* E9	MPTP-induced PD in C57BL/6 mice	15 days	Improved motor function, decreased levels of ROS, reduced dopaminergic cell loss and normalized gut bacterial composition

**Clinal study**	**Probiotic**	**Sample size (I/C)**	**Duration**	**Findings**
Cassani et al., 2011^83^	*L. casei* Shirota in fermented milk	40/0	5 weeks	Improved stool consistency, decreased bloating, abdominal pain, sensation of incomplete emptying
Barichella et al., 2016^93^	*B. breve*, *B. animalis* subsp. *lactis, E. faecium*, *L. acidophilus*, *L. delbrueckii* subsp. *bulgaricus, L. paracasei*, *L. plantarum*, *L. rhamnosus* GG and *S. salivarius* subsp. *thermophilus* in fermented milk	80/40	4 weeks	Increased complete bowel movements, decreased constipation
Georgescu et al., 2016^94^	*B. infantis* and *L. acidophilus*	20/20	12 weeks	Decreased abdominal pain and bloating
Borzabadi et al., 2018^95^	*B. bifidum, L. acidophilus, L. fermentum* and *L. reuteri*	25/25	12 weeks	Decreased levels of IL-1, IL-8 and TNF-α, increased levels of TGF-β and PPAR-γ
Tamtaji et al., 2018^96^	*B. bifidum, L. acidophilus, L. fermentum* and *L. reuteri*	30/30	12 weeks	Improved MDS-UPDRS score, decreased levels of hs-CRP, MDA, HOMA-IR and QUICKI, increased levels of GSH
Magistrelli et al., 2019^97^	*B. breve*, *B. animalis* subsp. *lactis*, *L. acidophilus*, *L. plantarum*, *L. rhamnosus* and *L. salivarius*	40/40	24 h	Decreased levels of TNF-α, IL-6, and IL-17A, increased levels of IL-4 and IL-10, inhibited inflammatory cytokines and ROS production
Ibrahim et al., 2020^98^	*B. infantis*, *B. longum*, *L. acidophilus*, *L. casei* and *L. lactis*	27/28	8 weeks	Improved bowel opening frequency and gastrointestinal motility, decreased constipation
Lu et al., 2021^99^	*L. plantarum* PS128	25/0	12 weeks	Improved MDS-UPDRS score, improved duration of ON periods and OFF periods
Tan et al., 2021^100^	*E. faecalis*, *E. faecium*, *B. bifidum*, *B. longum*, *L. acidophilus*, *L. paracasei*, *L. rhamnosus* and *L. reuteri*	34/38	4 weeks	Improved stool consistency, decreased constipation

I: intervention group; C: control group.

In most of the studies that found reduced levels of inflammatory factors, *L. plantarum* was used as a probiotic. *L. plantarum* has been reported in multiple studies as being associated with inhibition of neuroinflammation as well as oxidative stress.^85,87,101^ Two studies report changes in inflammatory factors without having used *L. plantarum*, but using a mixture of *B. bifidum*, *L. acidophilus*, *L. fermentum* and *L. reuteri*.^95,96^ An explanation for this could be that these probiotics alter the microbiota composition in a way that reduces the abundance of gram-negative bacteria, which consequently reduces the levels of LPS in the gut. A second explanation could be *L. reuteri*'s ability to express multiple proteins involved in redox processes.^102^ This might lead to reduced oxidative stress and reduced intestinal inflammation.

Though there is an increased abundance of *Lactobacillaceae* spp. in the gut of people with PD, which would logically be associated with higher SCFA production, in fact *lower* concentrations of fecal SCFAs were reported by various studies.^103–105^ A possible explanation for this may be that the abundance of several SCFA-producing bacterial genera is reduced in the context of PD, for which the relative increase of *Lactobacillaceae* abundance can insufficiently compensate. Inflammation-induced leakage of intestinal SCFAs into the blood plasma could be an alternative explanation for the lower concentrations of fecal SCFAs.^106,107^

In summary, many studies have investigated the consequences of *Lactobacillaceae*-containing probiotics on the symptoms of people with PD. In the next chapter, we will discuss the discrepancies and inconsistencies found in literature.

## Discrepancies in the relationship between *Lactobacillaceae* spp. and the motor and non-motor symptoms in PD

In previous chapters, it was mentioned that people with PD often show an increased abundance of *Lactobacillaceae* spp. in the gut, and two separate studies linked this to worse motor symptoms. Contrastingly, administration of *Lactobacillaceae*-containing probiotics resulted in improvement of different motor as well as non-motor symptoms in several murine and human studies. In addition to this, results in mouse studies as well as in clinical studies demonstrated a downregulation of pro-inflammatory factors and/or upregulation of anti-inflammatory factors after administration of *Lactobacillaceae*-containing probiotics, suggesting a beneficial effect.

A possible explanation for this apparent discrepancy may have to be sought at the species level.

Wearing-off has been associated with an increased intestinal abundance of *Lactobacillaceae* spp. in people with PD. Although more specific determination of *Lactobacillaceae* species was not performed in that study, it is conceivable that TDC-producing *Lactobacillaceae* species were responsible for the increased wearing-off. The increased abundance of *L. fermentum* that was found to be significantly associated with more severe motor symptoms in another study may likewise be explained by the TDC-producing character of this species. Conversely, upon reviewing studies on *Lactobacillaceae*-containing probiotics as a treatment for PD, it is noteworthy that most of the studies which found an improved motor function used the species *L. plantarum*, which is not known to be TDC-producing and of which anti-inflammatory properties have been discovered. The observation that *L. plantarum* specifically seems to result in improvements in symptomatology in neurodegenerative disease is shared by another recently published review article.^108^ In addition, there are two studies reporting improved motor function after use of probiotics containing other species of *Lactobacillaceae*. One study used *L. rhamnosus*,^92^ and the other used a mixture of *L. acidophilus, L. fermentum* and *L. reuteri.*^96^ While *L. rhamnosus*, *L. acidophilus* and *L. reuteri* do not contain the *tdc* gene,^97,109,110^ *L. fermentum* does. Thus, other mechanisms might well play a role.

Several studies using *Lactobacillaceae*-containing probiotics reported downregulation of pro-inflammatory factors and/or upregulation of anti-inflammatory factors after treatment. As *Lactobacillaceae* is one of the families increased in the gut of people with PD, this might seem inconsistent in the context of a hypothesis in which the altered gut microbiota trigger or facilitate the pathophysiology of PD via increased inflammation. However, for specific species (such as *L. plantarum*) the effects may well be different than for the bacterial family as a whole. Still, the exact role of *Lactobacillaceae* spp. specifically in intestinal inflammation has yet to be addressed in research.

In Table 2, a summary of the relationship between different *Lactobacillaceae* species to effects on PD symptoms is given.

**Table 2.** Summary of associations of various *Lactobacillaceae* spp. with PD symptoms in humans.

Suspected deleterious association with PD symptoms	Suspected beneficial association with PD symptoms	Uncertain association with PD symptoms
*L. brevis* ^a^ *L. curvatus* ^a^ *L. fermentum* ^b^	*L. plantarum* ^b^ *L. rhamnosus* ^c^ *L. casei* ^b^ (gastrointestinal symptoms only)	*L. acidophilus* ^d^ *L. delbrueckii* subsp. *bulgaricus* ^d^ *L. lactis* ^d^ *L. paracasei* ^d^ *L. reuteri* ^d^ *L. salivarius* ^d^

^a^ Assumed effect, based upon its ability to produce TDC.

^b^ Observed effect on symptoms in humans.

^c^ Assumed effect, based upon observed effects on symptoms in mice.

^d^ Has not been investigated separately from other species.

## Future research directions

There are three ways in which *Lactobacillaceae* spp. can be connected to motor symptoms: via the capability of some *Lactobacillaceae* species to produce the enzyme TDC, via the hormone ghrelin, which is negatively correlated with the abundance of *Lactobacillaceae*, and via dysbiosis-related intestinal inflammation. The relative contributions of each of these mechanisms could be a subject of further research.

Probiotics leading to improved motor function, as well as to decreased levels of pro-inflammatory factors and/or increased levels of anti-inflammatory factors mostly appear to include the *Lactobacillaceae* species *L. plantarum*. To further untangle the beneficial and detrimental effects of *Lactobacillaceae* in PD, future microbiome analyses in people with PD should as much as possible look at bacteria at the species level rather than merely at the family or genus level. Furthermore, in addition to the genetic level, more downstream (functional) analysis such as TDC activity could provide clarity regarding beneficial vs. detrimental intestinal bacteria in PD. More research is needed to understand the relationship between ghrelin and *Lactobacillaceae*, as well as to understand the possible place of ghrelin in the pathophysiology of PD. Additionally, further research into the causes and consequences of oxidative stress in the gut of people with PD and the relationship between different *Lactobacillaceae* species and their antioxidative properties might create more understanding on their roles in PD.

In conclusion, *Lactobacillaceae* might upon first glance appear to have a paradoxical role in PD. However, this is only apparent, as the effect seems to be dependent on the specific species of *Lactobacillaceae* involved. Pending further research, it is of great importance for clinicians to be careful and selective when prescribing certain *Lactobacillaceae*-containing probiotics. This review will hopefully serve as a solid foundation for further exploration of the use of probiotics in people with PD.
